# Multivariate Analysis Approaches for Dimension and Shape Discrimination of *Vitis vinifera* Varieties

**DOI:** 10.3390/plants10081528

**Published:** 2021-07-26

**Authors:** Muhammed Kupe, Bahadır Sayinci, Bünyamin Demir, Sezai Ercisli, Kürşat Alp Aslan, Muhammet Ali Gundesli, Mojmir Baron, Jiri Sochor

**Affiliations:** 1 Department of Horticulture, Faculty of Agriculture, Atatürk University, 25240 Erzurum, Turkey; muhammed.kupe@atauni.edu.tr (M.K.); sercisli@atauni.edu.tr (S.E.); 2 Department of Mechanical Engineering, Faculty of Engineering, Mersin University, 33340 Mersin, Turkey; bsayinci@mersin.edu.tr; 3 Department of Improvement and Genetics, Pistachio Research Institute, 27060 Gaziantep, Turkey; kursatalp0272@msn.com; 4 Department of Plant and Animal Production, Nurdağı Vocational School, Gaziantep University, 27310 Gaziantep, Turkey; maligun4646@gmail.com; 5 Department of Viticulture and Enology, Faculty of Horticulture, Mendel University, 69144 Lednice, Czech Republic; mojmirbaron@seznam.cz (M.B.); jiri.sochor@mendelu.cz (J.S.)

**Keywords:** surface area, projected area, physical characteristics, contour analysis, elliptic Fourier analysis

## Abstract

In this study, berry dimensions and shape traits, which are important for the design of the grape processing system and the classification of 10 different grape varieties grown in same ecological conditions (‘Ata Sarısı’, ‘Barış’, ‘Dımışkı’, ‘Hatun Parmağı’, ‘Helvani’, ‘Horoz Karası’, ‘Hönüsü’, ‘İtalia’, ‘Mevlana Sarısı’, and ‘Red Globe’) were determined; differences between the varieties were identified with the use of discriminant analysis. The largest grape varieties were identified as ‘Ata Sarısı’ and ‘Red Globe’. The ‘Red Globe’ and ‘Helvani’ varieties had geometrically sphere-like shape. The ‘Barış’ variety had the lowest size averages. According to elliptic Fourier analysis, the primary source of shape variation was ellipse and sphere-looking varieties. However, shape variation was seen due to the existence of a small number of drop-like varieties. According to discriminant analysis, shape differences of the varieties were defined by two discriminant functions. Based on these discriminant functions, the greatest classification performance was achieved for ‘Mevlana Sarısı’ and ‘Dımışkı’. In scatter plots, three shape definitions (sphere, ellipse, and drop) were made for grape varieties. Cluster analysis revealed 4 sub-groups. The first sub-group included the ‘Mevlana Sarısı’ variety; the second sub-group included the ‘Hönüsü’, ‘Hatun Parmağı’, ‘Dımışkı’, and ‘Horoz Karası’ varieties; the third sub-group included the ‘Ata Sarısı’ variety; the fourth sub-group included the ‘Barış’, ‘Helvani’, ‘İtalia’, and ‘Red Globe’ varieties. The variety in the first group had a geometrically ellipse-like shape, the largest length, and the smallest width. The size data were the smallest for the second sub-group. The third sub-group, with the ellipse-like shape, had the large size data. The grape varieties the closest to the sphere were classified in the fourth group, and these varieties had the large sizes.

## 1. Introduction

Horticulture sector, including viticulture, constitute an enormous income source for millions of farmers worldwide. All horticulture plants are an important source of nutrients, vitamins, minerals, dietary fibres, etc. They have been using in traditional medicine for a long time, because they contain health benefiting compounds. Horticulture plants are also widely used in the industry [1,2,3,4,5].

Viticulture is practiced in various parts of the world. It could be defined as an open-complex growing system, influenced by several factors, especially including the climate and soil conditions. On the other hand, the cultivar used in viticulture is one of the key issues for successful production. Therefore, before establishing a vineyard, the most appropriate cultivar should be selected for the place in which viticulture is to be practiced [6].

The shape of horticultural crops is the one of the most effective traits, and it is strongly related to the quality, quantity, and value of the crops. Shape is also important in the variety identification of horticultural crops as morphologically [5,7,8].

Moreover, recently agriculture system uses more mechanization and automation; shape is critical for the design of the machines. It is well known that the identification of the shapes of the horticulture crops has been performed for centuries by visual assessment alone, and the criteria for judgement have not been well defined. It has been considered challenging to transfer the classification of shapes from visual assessment to computerization, in part because it is difficult to verbally describe shapes in detail and in a standardized manner. The visual assessment approach is also labor-intensive, prone to human bias, and typically generates ordinal data less suitable for the most powerful, quantitative statistical models [9,10,11,12].

On the other hand, accurate and quantitative phenotypic data in plant breeding programmes is vital in breeding, to assess the performance of genotypes and to make selections. Fruit shape is important selection criterion for developing new varieties to meet specific market needs, in this context [5,13].

The traditional phenotyping of fruits relies on the human eye to assess most external fruit quality attributes, which is time-consuming and subjective. However, 3D imaging is a promising, high-throughput technique that allows multiple, external fruit quality attributes to be measured simultaneously [13,14,15].

More recently, several image-based phenotyping techniques have been implemented on fruits, including cherry laurel [16], strawberries [11,17,18], persimmons [19], cornelian cherry [15], hazelnut [10], etc. One promising method to describe fruit shape is based on elliptic Fourier descriptors (EFDs). All above studies characterized fruit shape diversity across cultivars using EFDs and PCA analysis, and they showed the usefulness of the EFDs with high accuracy.

Grape varieties are traditionally characterized by ampelographic traits, based on leaf, cluster and berry morphological characteristics [20,21,22,23,24,25,26,27,28], and they have diverse berry colour which varies from white to black with variations in purple, red, yellow, and green. Other variable berry characteristics for grapes are the fruit shapes (spherical (or round), oblate, ellipsoidal, obovoid, ellipsoidal elongated, ovoid, or oval) and cluster shapes (short conical, conical-shouldered, long conical, cylindrical, cylindrical-winged, and winged-double) [29]. Ampelography is currently keeping importance in grape variety identification and has proved useful for describing, and establishing relationships among, grape genetic resources. However, the method needs more time and experienced personnel. Same grape varieties also show berry size, detection, cluster compactness, etc. differences in different ecological conditions; additionally, developmental stage and canopy management affects traits in same variety berries [12,30,31,32]. In addition, the varieties that have irregular berry shapes make accurate identification difficult (by ampelographic classification). Moreover, in defining qualitative descriptors, such as shape and colour (where subjectivity often occurs from observer to observer) and the use of image analysis, eventually the conversion of qualitative data to quantitative data can be encouraged [33,34,35].

To characterize shapes in grape varieties, some other techniques have been conducted. These methods include the use of elliptic Fourier analysis (EFA) (that analyzes closed contours, immaterial of the size and configuration). The EFA method mathematically describes the overall shape of an object, by transforming the outline information into Fourier coefficients. So far, a few studies on grape leaf and berry shape characterization using EFA have been published [34,35].

Among horticultural crops, grapes are one of the most widely used species in the industry, along with citrus. Grapes are processed into fruit juice, dextrose, grape vinegar, grape molasses, and grape leather. Dried grapes (raisins) are both used in tables and in pastries (as an additive). Thus, it could be stated that the grape is a significant, directly processed foodstuff. In grape processing facilities, the washing, cleaning, separation, screening, classification, pressing, and packaging systems are designed based on the physical characteristics of the berries.

In the present study, the physical characteristics of 10 different grape varieties were determined, and the shape and dimensional traits were compared. Elliptic Fourier descriptors were used to put forth morphological differences, define shape geometries, and identify variations in the shape geometries of the grape varieties. Despite this, there are no reports on the application of image-based techniques, particularly EFA, to systematically establish shape descriptor states in Turkish main grape samples.

## 2. Materials and Methods

### 2.1. Sampling Location

Grape samples were harvested from the vineyards of Ahmet Münir Bilgen Production Facility of Pistachio Research Institute (Gaziantep) of Turkey in the 2020 growing season. The sampling vineyard is 11 years old, with a wired training system. Samples were taken to the Image Processing Laboratory of Advanced Technology Education Research and Application Center at Mersin University in the same day with frigorific vehicles (+4 °C).

### 2.2. Sample Imaging and Image Processing

**Figure 1:**
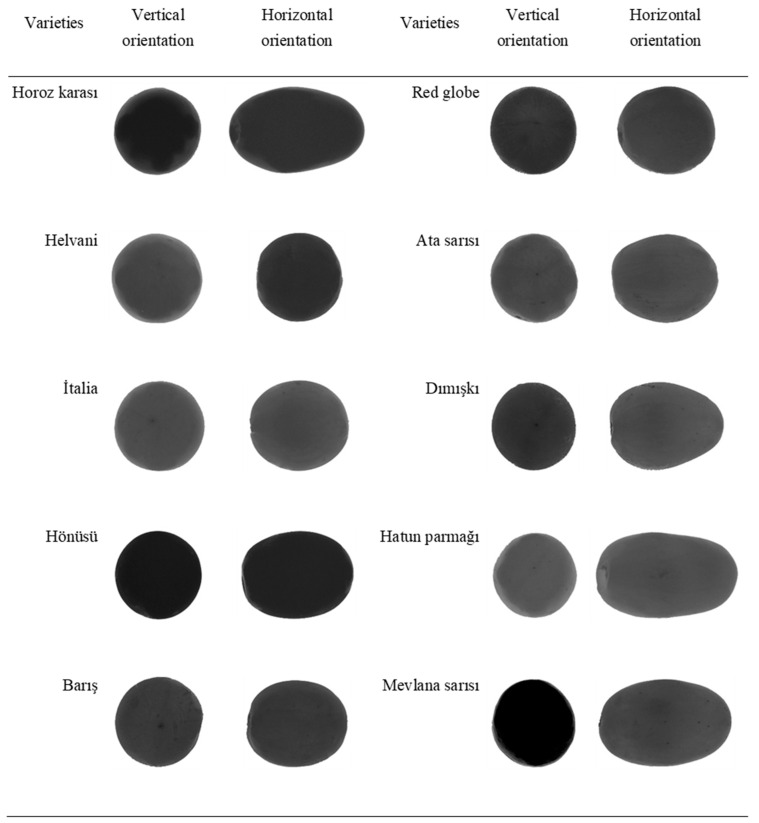
*Vitis vinifera* varieties.

**Figure 2:**
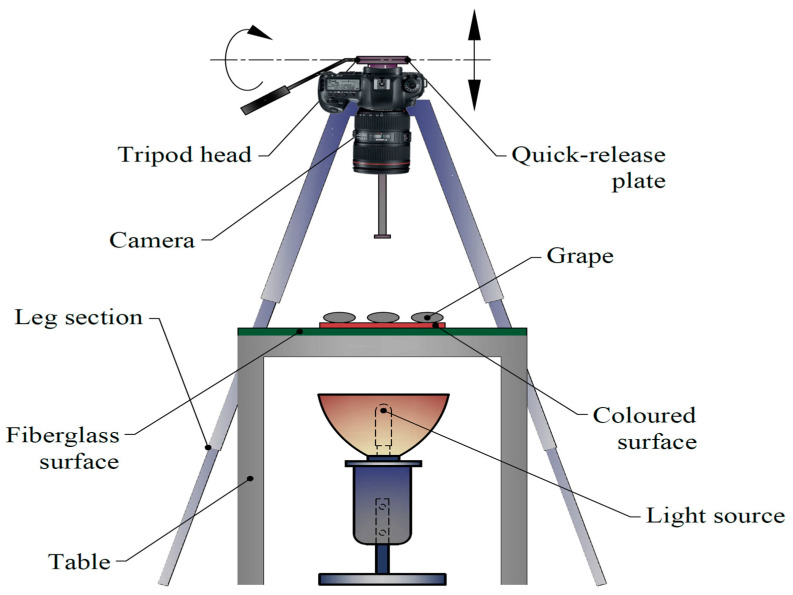
Image acquisition system.

As presented in Figure 1, 10 different grape varieties (‘Ata Sarısı’, ‘Barış’, ‘Dımışkı’, ‘Hatun Parmağı’, ‘Helvani’, Horoz Karası’, ‘Hönüsü’, ‘İtalia’, ‘Mevlana Sarısı’, and ‘Red Globe’) were used in this study. A randomly selected 40 samples were used for the image processing purposes of each variety. Sampled berries were imaged on white color fiberglass plates, supplemented with artificial lighting beneath for clear images [36] and a transparent surface to provide a contrast between plate and grape color. Samples were arranged in 4 × 5 matrix of 2 groups. Samples fixed with cylindrical plastic supports were imaged at horizontal and vertical orientations, with the use of Nikon D90 model digital camera and the resultant images were saved in *.tiff files. The schematic diagram of the imaging system, with a digital camera mounted on a tripod allowing imaging from 56 cm above the samples, is presented in Figure 2. An external shutter release button was used to prevent vibrations while imaging. A millimetric ruler was used to convert pixel units into metric units.

### 2.3. Dimension and Shape Traits

**Figure 3:**
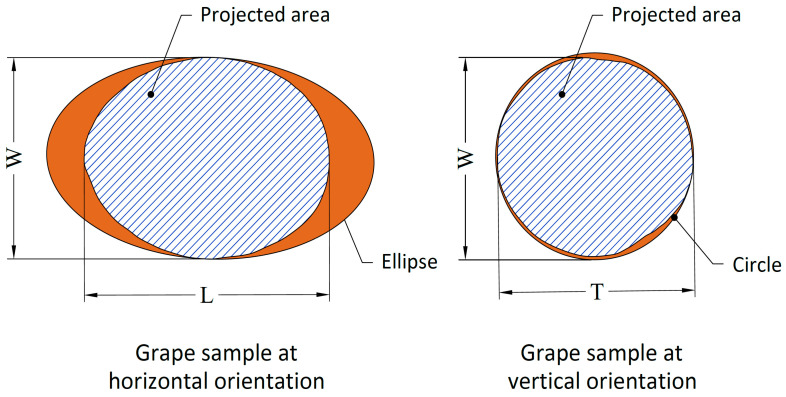
Size and area measurements of the grape varieties.

To determine the dimension and shape traits of the grape varieties, SigmaScan Pro v.5.0 software was used. Thresholding was applied to monochrome images in the range of 0–255, and dimension analysis was conducted automatically. The length (L, mm), width (W, mm), thickness (T, mm), projected area (PA, mm^2^), equivalent diameter (ED, mm), perimeter (P, mm), and circularity (C) values were measured. The measured dimension and shape traits are provided in Figure 3, and the equations used to calculate these values are provided in Table 1.

### 2.4. Elliptic Fourier Analysis

Image files of 40 berries of each variety were used in elliptic Fourier analysis (EFA) was conducted in 4 stages with the use of SHAPE (version 1.03) software [39]. Shape contours were defined in the first stage, x-y coordinates of the points on resultant curves were determined in the second stage, coordinates were converted into mathematical functions in the third stage, and function coefficients were determined over 20 harmonics in the fourth stage [40]. The harmonics each produced 4 Fourier coefficients (an, bn, cn, and dn) with an–bn representing the x coordinate and cn-dn representing the y coordinate of the curve [41,42].

Berry images were converted into 24-bit*.bmp files and shape for image processing. Shape data were gathered with the use of 4 different modules: image processing and shape contours were formed in the first module (ChainCoder), contour codes were normalized and elliptic Fourier descriptors were determined in the second module (Chc2Nef), PC analysis was conducted on resultant descriptors and PC scores were determined in the third module (PrinComp), and the shape variations of contours of berry shapes were visualized in the fourth module (PrinPrint).

### 2.5. Statistical Analyses

Variance analysis (ANOVA) was applied to data of the variables for physical characteristics with the use of SPSS 20.0 software, and Duncan’s test was used to compare significant means (*p* < 0.05).

PAST v.4.02 software was used for multivariate variance analysis (MANOVA), applied to the size and shape data of the grape varieties, and the contour codes were normalized with elliptic Fourier analysis (EFA).

The Hotelling’s pair-wise comparison test, including corrected Bonferroni values and Mahalanobis distances, was used to explain the size and shape differences of the grape varieties.

Discriminant analysis was conducted with the use of the size and shape data, and PC scores, to identify the functions yielding the size and shape differences of grape varieties and similarity relationships, were presented on a scatter plot. In addition, such similarities were also revealed with the hierarchical clustering analysis using the Euclidean similarity index, and the size and shape differences/similarities, using the discriminant scores obtained from both EFA and size data between the grape varieties, were presented on a dendrogram.

## 3. Results and Discussion

The greatest projected area, equivalent diameter, and perimeter values at both the horizontal and vertical orientations were obtained from ‘Ata Sarısı’ and ‘Red Globe’ varieties (Table 2). On the other hand, the lowest values were obtained from the ‘Barış’ variety at the horizontal orientation and from the ‘Hatun Parmağı’ variety at the vertical orientation. A circularity value of 1 represents a full circle. Therefore, the ‘Helvani’ and ‘İtalia’ varieties at the horizontal, and the ‘Helvani’ and ‘Hönüsü’ varieties at the vertical orientation, had the closest shape to a circle. Grape breeding in Turkey is, in general, focused on the development of table grapes with desirable traits such as large berries and bunch size [20,24]. In fact, the grape berry and bunch characteristics are widely investigated in grape-growing countries, and all studies revealed significant differences among the studied varieties for the measured characters [22,23,25,28]. Abiri et al. [43] used 55 grape varieties in their morphological analysis and reported variable berry dimensions. They found berry lengths between 12.32–31.85 mm, and berry widths between 10.55–23.45 mm. Khadivi-Khub et al. [44] reported berry lengths and widths between 10.00–34.00 mm and 7.00–23.00 mm, respectively. Vafaee et al. [45] reported the range of 9.80–30.30 mm for berry length.

Abiri et al. [43] found quite variable berry shapes in grape varieties in Iran and among 55 investigated varieties, 7 varieties were found to have an oblong berry shape, 8 genotypes had a narrow-elliptic shape, 13 varieties had an elliptic shape, 16 varieties had a round shape, one variety had an oblate shape, 5 varieties had ovate shapes, 4 varieties had obtuse-ovate shapes, and one variety had an obovate berry shape. Khadivi- Khub et al. [44] observed three types of berry shape: oblong, elliptic, and round in grape cultivars grown in Iran.

Previously, Ekhvaia and Akhalkatsi [46], Leão et al. [47], and Esgici et al. [48] studied grape genotypes based on berry dimensions and reported high-variability among varieties. Kok et al. [30] investigated the dimensional attributes of 8 grape cultivars in western Turkey and found that berry dimensions quit being variable among 8 grape cultivars. Previous studies indicated that grape berry dimensions are cultivar-dependent, yet it is affected by numerous factors, including gibberellin treatments, canopy, girdling, soil type, irrigation, rootstock, weather, etc. [49,50,51].

‘Horoz Karası’ and ‘Mevlana Sarısı’ varieties had greater length and elongation ratios than the other varieties (Table 3). Geometric mean diameter, surface area, and volume variables are used to compare fruit size. Based on these values, ‘Red Globe’ was identified as the largest variety. In terms of sphericity, ‘Helvani’ and ‘Red Globe’ had the closest shape to a sphere; thusly, elongation ratios prove that finding. ‘Barış’ and ‘Hatun Parmağı’ varieties had the lowest surface areas. Bart-Plange et al. [52] indicated that the heat transfer rate of a material relied on its surface area. Increasing heat transfer rates were reported with decreasing volume/surface area ratios, and such criteria was indicated as an important parameter characterizing the drying duration.

The unique nature and attractive appearance of the horticulture crops, including fruits, vegetables, and grapes (which are used both for fresh and industrial purposes) has attracted more and more consumers’ attention in recent years [53,54]. Among external quality parameters, fruit shape, related to dimensions, is an important quantitative trait closely related to the fruit quality [5]. Fruit shape is a widely searched parameter in horticulture, and this information is important in selecting appropriate parents and in developing the most appropriate strategy for breeding horticulture crops, both for table and industrial use. Horticulture plants are, in general, grown in an open field and their morphology is affected by environmental conditions, e.g., weather, soil, cultivation, and management [55,56,57]; however, the phenotypic variations for many horticulture crops are mainly affected by genetic background (parents) [58,59,60,61].

In general, fruit morphological approaches for external characteristics (in most of the horticultural crops) are used, and often rely, on the human eye to make categorical assessments. However, fruit shape is an inherently multi-dimensional, continuously variable trait and is not adequately described by a single categorical or quantitative feature. Thus, more recently, some digital image approaches were developed to eliminate human mistakes. Those approaches make fruit shape categories human-recognizable. These methods are based on select quantitative features, extracted from multiple morphometric analyses, that are the best fit for genetic dissection and analysis [10,11,16,35].

The structure matrix, obtained as a result of the discrimination analysis, is given in Table 4. The size and shape variables that were not included in the discrimination functions were excluded from the analysis. The first and second discriminant functions have the largest correlations. The first function divides grape varieties into sphericity and elongation characteristics. The second function reveals the differences in the size characteristics of grape varieties. The other, largest correlations have the fifth and seventh functions. These functions distinguish grape varieties according to their circularity.

In Table 5, the size and shape traits of grape varieties were classified with 9 discrimination functions. The first function explains 65.7% of the total variance, and the second function explains 12.3%. According to the canonical correlation coefficients, the difference between the groups can be explained by 95.8% of the first function and 82.4% of the second function. According to the results of MANOVA and Hotelling’s pairwise comparison, the grape varieties differ from each other, according to the size and shape traits. However, in paired comparisons, the affinities between the varieties can be examined according to Mahalanobis distances. The smaller the distance value, the higher the similarity rate between varieties. The classification performance of the discrimination functions is 80.8%. Although there are significant differences between the varieties, according to their size and shape traits, the varieties with similar characteristics are clearly seen in the table. For example, the correct classification rate of the ‘Mevlana yellow’ variety, by the discrimination function, is 70%. However, this grape variety has similar characteristics with the ‘Horoz Karası’ and ‘Hatun Parmağı’ varieties. It is seen that the Mahalanobis distances of both varieties are smaller than the other varieties.

**Figure 4:**
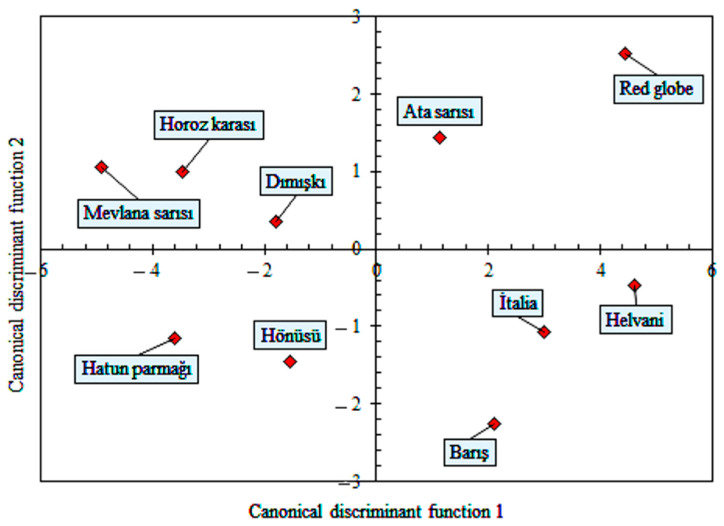
Biplot charts from the linear discriminant analysis of 10 grape varieties, based on the size and shape data, measured or calculated using image processing method.

According to Figure 4, the ‘Red Globe’ and ‘Helvani’ varieties, placed on the right side of the function 1, had the largest sphericity. On the other hand, ‘Mevlana Sarısı’ had the smallest sphericity. Sayinci et al. [16] reported average sphericity of cherry laurel genotypes as 94.3%. Compared to the general average, the sphericity of cherry laurel genotypes is higher than that of grape varieties. The maximum elongation value had negative correlation with the discrimination function 1. Therefore, all grape varieties (‘Mevlana Sarısı’, ‘Hatun parçağı’, ‘Horoz Karası’, ‘Dımışkı’, and ‘Hönüsü’) to the left of the function 1 axis had greater elongation values. The correlation between the discriminant function 2 and the size of the grape varieties is positive. Therefore, the size data of the grape varieties above the function 2 axis are larger than the others.

**Figure 5:**
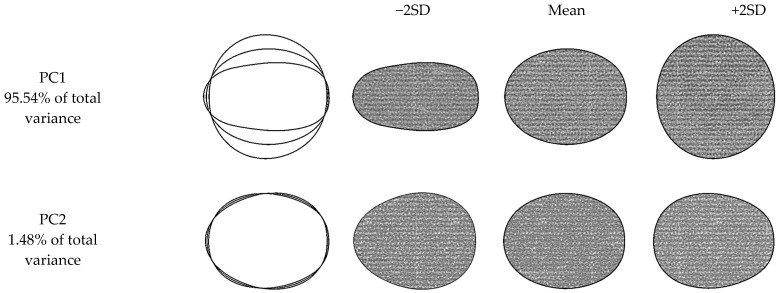
Principal components (PCs) for shape variables of grape varieties, based on a principal component analysis of 800 fruit outlines. From left to right, the outlines show the principal component scores corresponding to: mean −2 standard deviations, mean, and mean +2 standard deviations.

The results of elliptic Fourier analysis are presented in Figure 5. Two principal components explained 97.02% of the total variation in the shapes of the grape varieties. PC1 explained 95.54% of the total variation. Shape differences, the majority of which were explained by PC1, were mainly attributed to ellipse and sphere-looking varieties. Similar findings were also reported by Bodor et al. [35] for five different grape genotypes. PC2 explained only 1.48% of the total variation in the shapes of the grape varieties. Considering the shape variation of PC2, it was observed that there were drop-like varieties, apart from ellipse and sphere geometries.

In the present study, berry traits proved useful in assessing the diversity and relationships of Turkish grape varieties as well-known grape genetic resources. The current study revealed considerable diversity in some berry characteristics of the grape varieties. The potential use of Turkey’s grape varieties as genetic resources in breeding programmes was highlighted for further investigation.

Principal component analysis is one of the most important and powerful methods in both the morphometric and chemometric characterization of grape varieties, as well as PCA revealing dimension and shape differences among grape varieties [25,26,47]. Abiri et al. [43] used PCA to establish the relationships among 55 grape cultivars in Iran and showed the method effective to grouping grape cultivars. The present findings, in some cases, corresponded with the previous results in the grape PCA analysis [44,45,46,47,48].

Principal component analysis was also used in put forth dimension and shape differences in walnuts [13], oranges [7], and beans [36].

The results of multivariate variance analysis, conducted with the use of elliptic Fourier component scores and results of canonic discriminant analysis, are provided in Table 6. There are significant shape differences between the varieties. Two functions, discriminating varieties based on shape differences, explained 100% of total variation. The classification performance of the discriminant functions was identified as 56.0%. This result shows that the canonical functions are not able to classify grape varieties properly. However, considering that the shape geometries of the grape varieties are only ellipse, sphere, and drop-like, the success of the canonical functions in classification is quite high. This success in classification depends on the scores of the first two principal components, which explain the shape differences between the grape varieties. By using these component scores, the discrimination functions are obtained. While the first discriminant function classifies grape varieties according to ellipse and sphere geometry, the second discrimination function classifies them according to the drop appearance. The greatest classification performance was achieved for the ‘Mevlana Sarısı’ and ‘Dımışkı’ varieties. Pairwise comparison tests revealed that the shape difference between ‘Red Globe’ and ‘İtalia’ varieties was not significant. The shape differences between the other varieties were explained with Mahalanobis distances, and values closer to zero indicated increasing similarity between the varieties.

**Figure 6:**
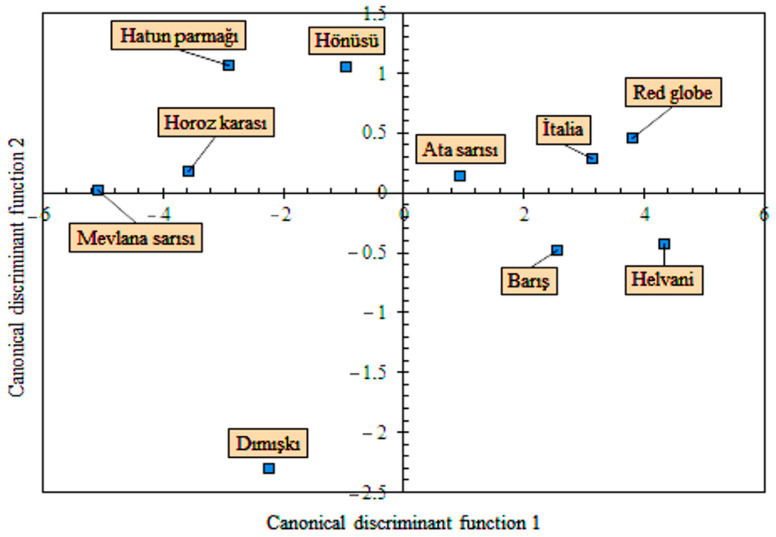
Biplot charts from linear discriminant analysis of 10 grape varieties, based on two principal components of shape variables, derived from elliptic Fourier data of 400 fruit outlines (the locations of the varieties on the chart show their own group centroid).

A scatter plot is presented in Figure 6 for the discriminant scores of the grape varieties. While the ‘Red Globe’ and ‘Helvani’ varieties on the right side of the Function 1 axis had a sphere-like shape, the ‘Mevlana Sarısı’ on the left side had a geometrically ellipse shape. Among the varieties, ‘Dımışkı’ was placed on a different coordinate. It was placed beneath the Function 2 axis and had a drop-like shape.

**Figure 7:**
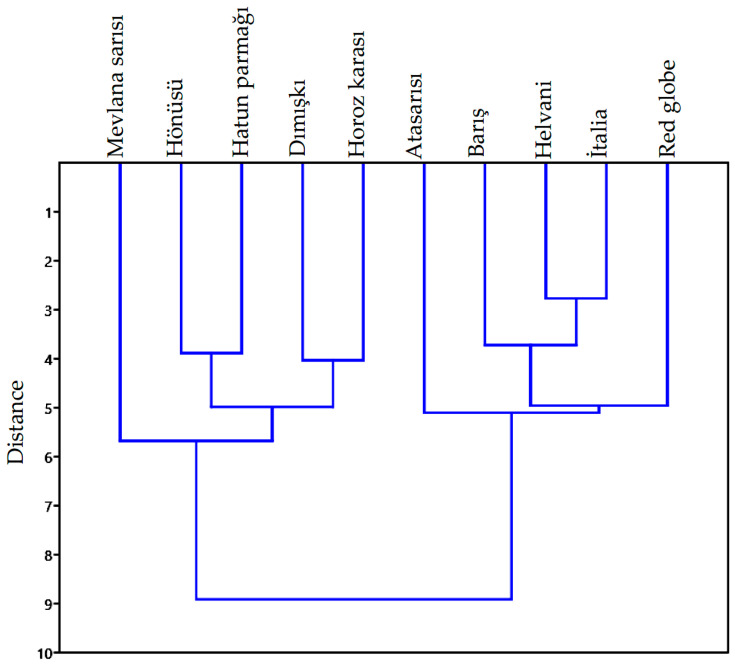
Dendrogram of 10 grape varieties for the discriminant scores obtained from the size and shape data, and principle component scores of the elliptic Fourier analysis using hierarchical cluster analysis (between-group linkage method and Euclidean distance).

The results of the cluster analysis are presented in Figure 7. According to cluster analysis, grape varieties were gathered under two main groups, in terms of size and shape. Each main group had two sub-groups (a total of 4 sub-groups): the first sub-group included the ‘Mevlana Sarısı’ cultivar; the second sub-group included the ‘Hönüsü’, ‘Hatun Parmağı’, ‘Dımışkı’, and ‘Horoz Karası’ varieties; the third sub-group included the ‘Ata Sarısı’ variety; the fourth sub-group included the ‘Barış’, ‘Helvani’, ‘İtalia’, and ‘Red Globe’ varieties. The variety in the first group had a geometrically ellipse-like shape and the lowest sphericity. The size data of the varieties in the second group were smaller than those of the others. The geometric shape of the variety in the third sub-group was similar to the ellipse and had the large size data. A definition of a sphere was made for the shape of the varieties in the fourth sub-group, and these varieties had the highest size average.

## 4. Conclusions

The majority of harvest machines exhibit image-processing-based operation. Fully automated systems include identification, cut/pull off, and transfer processes, based on color, dimension, and shape variables. Present findings constitute a significant source of data for the design of grape processing technologies. Shape traits, determined based on the closed contour geometry of the grape varieties, also play an important role in monitoring possible mutant changes to be encountered, due to production conditions. Among the present grape varieties, there were small, medium, large, and very large varieties. The geometrical shape of grape is generally ellipse. However, sphere-like geometries generated a shape variation. Accordingly, an ellipse could be defined for ‘Mevlana Sarısı’, and a sphere could be defined for ‘Helvani’ and ‘Red Globe’ varieties. The analysis conducted, with the use of elliptic Fourier descriptors, revealed that there were also drop-like varieties. According to the discriminant analysis, the shape differences of the varieties were explained by two discriminant functions. According to the pairwise comparison test, there were not significant shape differences between the ‘Red Globe’ and ‘İtalia’ varieties. On the other hand, there were significant shape differences between the other varieties. However, the use of more than one variety may offer some advantages in the design of food processing systems. In this sense, the shape similarities of the varieties could be assessed with the use of Mahalanobis distances. In scatter plots, generated based on discriminant functions, contour changes were re-evaluated to describe the geometric shapes of the varieties, and matching was made with picture images to prove the shapes. Accordingly, grape varieties were defined with three geometric shapes: sphere, ellipse, and drop. Cluster analysis revealed 4 sub-groups, and these sub-groups could further be divided into sub-sub-groups. The operational performance of the product classification system relies on product dimensions and shape traits. Therefore, alternative products should be designed for the classification of different varieties in the same system.

## Figures and Tables

**Table 1:** Equations used for size and shape traits of the grape varieties.

Size and Shape Traits	Equations	References
Maximum elongation ($E_{h}$)	$E_{h}=L/W$	[15]
Minimum elongation ($E_{v}$)	$E_{v}=W/T$	[15]
Geometric mean diameter (*D_g_*, mm)	$D_{g}=\sqrt[{3}]{\left(L\cdot W\cdot T\right)}$	[37]
Sphericity (*φ*, %)	$\varphi =\left(D_{g}/L\right)\cdot 100$	[38]
Volume (*V*, mm^3^)	V=(π/6)·(L·W·T)	Ellipse volume
Surface area (*SA*, mm^2^)	$SA=\pi \cdot D_{g}^{2}$	[16]
Circularity (*C*)	$C=4\cdot \pi \cdot PA/P^{2}$	[13]

**Table 2:** Basic size and shape traits measured at horizontal and vertical orientations.

Varieties	Horizontal Orientation				Vertical Orientation			
	Projected Area (mm^2^)	Equivalent Diameter (mm)	Perimeter (mm)	Circularity	Projected Area (mm^2^)	Equivalent Diameter (mm)	Perimeter (mm)	Circularity
Ata Sarısı	611.1 ± 56.0 a *	27.87 ± 1.26 a	95.33 ± 4.60 ab	0.844 ± 0.019 b	499.9 ± 46.3 b	25.20 ± 1.16 b	87.30 ± 4.51 b	0.824 ± 0.050 d
Barış	430.0 ± 55.0 e	23.35 ± 1.49 e	80.02 ± 6.04 e	0.842 ± 0.032 b	376.4 ± 44.7 de	21.85 ± 1.29 de	74.54 ± 4.58 d	0.849 ± 0.029 bc
Dımışkı	527.8 ± 36.4 c	25.91 ± 0.89 c	90.71 ± 3.52 c	0.806 ± 0.026 c	390.1 ± 27.3 d	22.27 ± 0.77 d	75.56 ± 2.90 d	0.858 ± 0.019 b
Hatun Parmağı	467.4 ± 40.0 d	24.37 ± 1.03 d	84.91 ± 4.07 d	0.814 ± 0.020 c	306.0 ± 25.3 g	19.72 ± 0.81 f	67.47 ± 3.04 f	0.844 ± 0.031 bcd
Helvani	492.7 ± 34.4 d	25.03 ± 0.87 d	84.47 ± 3.04 d	0.867 ± 0.014 a	474.9 ± 37.3 b	24.57 ± 0.96 b	82.01 ± 3.18 c	0.886 ± 0.008 a
Horoz karası	585.0 ± 48.4 ab	27.27 ± 1.14 ab	94.69 ± 3.95 ab	0.819 ± 0.014 c	390.3 ± 38.5 d	22.27 ± 1.13 d	75.64 ± 4.12 d	0.856 ± 0.042 b
Hönüsü	483.6 ± 35.6 d	24.80 ± 0.91 d	84.58 ± 3.36 d	0.849 ± 0.013 b	356.0 ± 21.9 ef	21.28 ± 0.65 e	71.06 ± 2.15 e	0.885 ± 0.007 a
İtalia	479.7 ± 51.0 d	24.68 ± 1.30 d	83.21 ± 4.29 d	0.868 ± 0.016 a	433.3 ± 45.8 c	23.46 ± 1.22 c	79.80 ± 3.70 c	0.853 ± 0.032 b
Mevlana sarısı	550.2 ± 55.7 bc	26.44 ± 1.34 bc	93.76 ± 5.16 bc	0.785 ± 0.021 d	329.8 ± 34.5 fg	20.47 ± 1.06 f	70.82 ± 4.50 e	0.827 ± 0.049 cd
Red globe	615.1 ± 68.6 a	27.94 ± 1.53 a	97.20 ± 5.57 a	0.818 ± 0.050 c	577.2 ± 73.1 a	27.06 ± 1.69 a	93.71 ± 7.05 a	0.825 ± 0.039 cd
Mean ± SD	524.3 ± 77.9	25.77 ± 1.91	88.89 ± 7.29	0.831 ± 0.036	413.4 ± 89.5	22.81 ± 2.42	77.79 ± 8.71	0.851 ± 0.040
Min–max	327.6–822.0	20.42–32.35	68.39–112.74	0.654–0.891	254.7–789.6	18.01–31.71	61.81–111.2	0.667–0.897

* Means followed by the same letter in the same column are not significantly different based on Duncan’s test at 5% significance level.

**Table 3:** Characteristic size and shape traits of the grape varieties.

Varieties	Length (mm)	Width (mm)	Thickness (mm)	Geometric Mean Diameter (mm)	Sphericity (%)	Max Elongation	Min Elongation	Surface Area (cm^2^)	Volume (cm^3^)
Ata Sarısı	30.87 ± 1.78 b *	25.21 ± 1.36 b	25.60 ± 1.25 b	27.10 ± 1.19 b	87.9 ± 3.2 e	1.227 ± 0.076 f	1.046 ± 0.018 bc	23.11 ± 2.06 b	10.476 ± 1.413 b
Barış	24.96 ± 1.81 e	21.94 ± 1.38 e	21.96 ± 1.36 f	22.90 ± 1.40 g	91.9 ± 2.7 d	1.139 ± 0.056 g	1.037 ± 0.021 cd	16.54 ± 2.02 h	6.360 ± 1.169 gh
Dımışkı	30.83 ± 1.64 b	21.98 ± 0.86 e	22.70 ± 0.80 e	24.86 ± 0.79 d	80.8 ± 3.2 g	1.405 ± 0.089 d	1.038 ± 0.019 cd	19.44 ± 1.24 de	8.070 ± 0.779 de
Hatun parmağı	29.28 ± 1.81 c	19.66 ± 0.82 h	19.98 ± 1.05 h	22.56 ± 0.87 g	77.2 ± 3.3 h	1.491 ± 0.093 c	1.049 ± 0.028 b	16.01 ± 1.24 h	6.035 ± 0.706 h
Helvani	25.94 ± 1.04 d	24.67 ± 0.99 c	24.65 ± 1.06 c	25.07 ± 0.92 d	96.7 ± 1.9 a	1.052 ± 0.030 i	1.029 ± 0.016 de	19.78 ± 1.47 d	8.288 ± 0.926 d
Horoz karası	33.77 ± 1.55 a	22.00 ± 1.02 e	22.70 ± 1.35 e	25.64 ± 1.13 c	76.0 ± 2.3 i	1.536 ± 0.065 b	1.041 ± 0.023 bc	20.69 ± 1.80 c	8.874 ± 1.142 c
Hönüsü	28.61 ± 1.41 c	21.19 ± 0.81 f	21.41 ± 0.80 g	23.49 ± 0.77 f	82.2 ± 2.5 f	1.351 ± 0.067 e	1.045 ± 0.023 bc	17.36 ± 1.13 g	6.809 ± 0.666 g
İtalia	26.11 ± 1.39 d	23.45 ± 1.32 d	23.58 ± 1.23 d	24.34 ± 1.23 e	93.3 ± 2.1 c	1.114 ± 0.039 g	1.026 ± 0.015 e	18.66 ± 1.90 ef	7.611 ± 1.176 ef
Mevlana sarısı	33.77 ± 2.20 a	20.17 ± 1.10 g	20.92 ± 1.16 g	24.23 ± 1.15 e	71.9 ± 2.7 j	1.676 ± 0.097 a	1.063 ± 0.031 a	18.48 ± 1.77 f	7.495 ± 1.082 f
Red Globe	29.29 ± 1.59 c	27.08 ± 1.61 a	27.22 ± 1.97 a	27.83 ± 1.61 a	95.1 ± 2.3 b	1.083 ± 0.042 h	1.042 ± 0.025 bc	24.42 ± 2.88 a	11.404 ± 2.06 a
Mean ± SD	29.34 ± 3.35	22.73 ± 2.50	23.07 ± 2.45	24.80 ± 1.97	85.3 ± 8.8	1.307 ± 0.217	1.042 ± 0.024	19.45 ± 3.14	8.142 ± 2.009
Min–max	21.06–39.33	17.99–32.07	18.22–31.75	20.09–32.3	65.4–99.1	1.011–1.961	1.003–1.155	12.68–32.77	4.245–17.641

* Means followed by the same letter in the same column are not significantly different based on Duncan’s test at 5% significance level.

**Table 4:** The correlations between discriminating variables and standardized canonical discriminant functions.

Size and Shape Traits	Functions								
	1	2	3	4	5	6	7	8	9
Sphericity	0.942 *	−0.207	−0.049	−0.070	−0.039	0.053	0.044	−0.152	−0.041
Max elongation	−0.878 *	0.277	0.216	0.215	0.027	−0.088	0.014	0.140	0.062
Perimeter at horizontal	−0.077	0.881 *	−0.098	−0.041	−0.057	0.121	−0.225	0.195	0.283
Projected area at horizontal	−0.009	0.832 *	−0.18	−0.027	0.070	0.317	−0.363	0.107	0.133
Length	−0.393	0.817 *	−0.132	0.155	0.042	0.174	−0.246	0.146	0.141
Perimeter at vertical	0.420	0.799 *	−0.169	−0.133	−0.072	0.321	−0.021	0.103	−0.138
Thickness	0.394	0.726 *	−0.246	−0.033	0.044	0.176	−0.116	−0.033	0.119
Width	0.481	0.704 *	−0.226	−0.078	0.088	0.215	−0.329	−0.033	0.043
Circularity at horizontal	0.219	−0.337	−0.221	0.108	0.502 *	0.488	−0.226	−0.258	−0.398
Circularity at vertical	0.021	−0.253	−0.173	0.185	0.405	−0.416	−0.572 *	−0.001	0.396

* The largest absolute correlation between each variable and any discriminant function.

**Table 5:** The results of the discriminant analysis and pairwise comparisons.

A. Canonical Discriminant Functions (Computed in SPSS ver. 20)										
Functions	Eigenvalue		% of Variance		Cumulative, %			Canonical Correlation		
1	11.302		65.7		65.7			0.958		
2	2.110		12.3		77.9			0.824		
3	1.503		8.7		86.7			0.775		
4	1.298		7.5		94.2			0.752		
5	0.501		2.9		97.1			0.578		
6	0.366		2.1		99.2			0.517		
7	0.071		0.4		99.6			0.257		
8	0.054		0.3		100.0			0.227		
9	0.006		0.0		100.0			0.079		
B. MANOVA Results (computed in PAST ver. 4.05)										
Statistics	Value		Hypothesis df		Error df		F Value	p (Sigma)		
Wilks’ lambda	0.001952		90		2594		43.5	0.0000 **		
Pillai trace	3.488		90		3501		24.61	0.0000 **		
C. Hotelling’s Pairwise Comparisons. (Bonferroni Corrected p Values in Upper Triangle; Mahalanobis Distances in Lower Triangle) (Computed in PAST ver. 4.05)*										
Varieties	Horoz karası	Helvani	İtalia	Hönüsü	Barış	Red Globe	Ata Sarısı	Dımışkı	Hatun Parmağı	Mevlana sarısı
Horoz karası		7.5E−39 **	1.0E−3 **	6.1E−20 **	4.0E−33 **	6.6E−40 **	7.8E−26 **	2.7E−11 **	5.8E−23 **	8.7E−19 **
Helvani	72.5		2.6E−09 **	3.6E−33 **	2.1E−20 **	8.9E−21 **	4.5E−26 **	5.5E−34 **	2.6E−40 **	4.7E−43 **
İtalia	50.2	6.2		1.2E−24 **	4.8E−11 **	2.7E−22 **	7.7E−18 **	1.1E−27 **	5.4E−34 **	8.5E−39 **
Hönüsü	17.4	48.3	25.4		8.3E−24 **	1.6E−35 **	5.8E−21 **	2.3E−12 **	5.3E−15 **	2.9E−28 **
Barış	48.1	18.1	7.6	23.8		4.7E−28 **	2.6E−22 **	2.7E−26 **	4.8E−31 **	8.3E−38 **
Red Globe	78.2	18.6	21.1	57.3	33.0		1.8E−21 **	2.3E−34 **	4.0E−40 **	4.2E−43 **
Ata Sarısı	27.9	28.4	14.5	18.9	21.1	19.7		4.1E−19 **	1.2E−29 **	1.6E−34 **
Dımışkı	7.8	51.2	32.1	8.7	28.9	52.7	16.2		9.6E−21 **	3.5E−26 **
Hatun Parmağı	22.3	80.4	51.2	11.2	41.3	79.4	37.3	18.6		2.5E−19 **
Mevlana sarısı	15.8	97.4	72.3	33.6	67.4	97.8	53.3	28.6	16.5	
D. % Classification Performance (80.8% of Original Grouped Cases Were Correctly Classified) (Computed in SPSS ver. 20.0)										
Varieties	Horoz karası	Helvani	İtalia	Hönüsü	Barış	Red Globe	Ata Sarısı	Dımışkı	Hatun Parmağı	Mevlana sarısı
Horoz karası	87.5			2.5				5		5
Helvani		87.5	10		2.5					
İtalia		10	70		15	2.5	2.5			
Hönüsü				90	2.5			2.5	5	
Barış		5	15	5	70	2.5	2.5			
Red Globe		7.5	2.5			85	5			
Ata Sarısı		2.5	5			5	87.5			
Dımışkı	12.5		2.5	10			2.5	72.5		
Hatun Parmağı				2.5				5	87.5	5
Mevlana sarısı	12.5								17.5	70

** The grape varieties are significantly different in terms of the size and shape traits (*p* < 0.01 significant).

**Table 6:** The results of the discriminant analysis and pairwise comparisons.

A. Canonical Discriminant Functions (Computed in SPSS ver. 20)										
Functions	Eigenvalue		% of variance		Cumulative %			Canonical correlation		
1	10.612		92.6		92.6			0.956		
2	0.853		7.4		100.0			0.679		
B. MANOVA Results (Computed in PAST ver. 4.05)										
Statistics	Value		Hypothesis df		Error df		F value	p (Sigma)		
Wilks’ lambda	0.04647		18		778		157.3	0.0000		
Pillai trace	1.374		18		780		95.17	0.0000		
C. Hotelling’s Pairwise Comparisons. (Bonferroni corrected p values in upper triangle; Mahalanobis distances in lower triangle) (computed in PAST ver. 4.05) *										
Varieties	Horoz karası	Helvani	İtalia	Hönüsü	Barış	Red Globe	Ata Sarısı	Dımışkı	Hatun Parmağı	Mevlana sarısı
Horoz karası		1.73E−46	2.35E−41	3.66E−17	1.08E−38	2.60E−44	1.23E−29	1.19E−17	1.72E−03	6.56E−07
Helvani	62.57		1.06E−05	3.30E−35	5.60E−09	4.99E−03	2.91E−22	6.29E−42	2.33E−44	5.07E−52
İtalia	45.00	1.90		2.14E−27	1.05E−02	5.42E−01 *	1.47E−12	7.28E−38	1.66E−38	9.75E−48
Hönüsü	7.61	29.96	17.32		4.40E−25	2.35E−31	9.74E−12	1.37E−23	1.24E−10	4.70E−28
Barış	37.80	3.15	0.95	14.58		2.68E−07	1.68E−08	2.61E−33	2.64E−36	2.01E−45
Red Globe	54.46	1.04	0.47 *	22.99	2.48		3.99E−18	3.97E−41	1.43E−41	3.10E−50
Ata Sarısı	20.36	11.71	4.84	4.42	2.95	8.29		1.61E−26	3.87E−26	4.09E−38
Dımışkı	7.95	46.70	35.79	13.00	26.32	44.34	16.23		2.46E−22	4.91E−24
Hatun Parmağı	1.18	54.62	37.34	3.89	32.25	45.63	15.78	11.78		3.07E−14
Mevlana sarısı	2.33	88.64	67.73	18.17	58.47	79.27	36.38	13.46	5.76	
D. Classification performance (56.0% of original grouped cases were correctly classified) (computed in SPSS ver. 20.0)										
Varieties	Horoz karası	Helvani	İtalia	Hönüsü	Barış	Red Globe	Ata Sarısı	Dımışkı	Hatun Parmağı	Mevlana sarısı
Horoz karası	37.5	0	0	5.0	0	0	0	2.5	35.0	20.0
Helvani	0	70.0	7.5	0	2.5	20.0	0	0	0	0
İtalia	0	15.0	32.5	0	17.5	22.5	12.5	0	0	0
Hönüsü	0	0	0	67.5	0	0	10.0	7.5	15.0	0
Barış	0	15	22.5	0	40.0	2.5	20.0	0	0	0
Red Globe	0	27.5	25.0	0	7.5	40.0	0	0	0	0
Ata Sarısı	0	5.0	2.5	22.5	17.5	0	52.5	0	0	0
Dımışkı	10.0	0	0	0	0	0	2.5	87.5	0	0
Hatun Parmağı	25.0	0	0	22.5	0	0	0	0	45.0	7.5
Mevlana sarısı	12.5	0	0	0	0	0	0	0	0	87.5

* The grape varieties shown in color are not significantly different in terms of shape (*p* > 0.05 insignificant).

## Data Availability

All-new research data were presented in this contribution.

## References

[1] Ercisli S., Esitken A., Cangi R., Sahin F. (2003). Adventitious root formation of kiwifruit in relation to sampling date, IBA and *Agrobacterium rubi* inoculation. Plant Growth Regul..

[2] Sansavini S. (2006). The role of research and technology in shaping a sustainable fruit industry: European advances and prospects. Rev. Bras. Frutic..

[3] Dogan H., Ercisli S., Jurikova T., Temim E., Leto A., Hadziabulic A., Tosun M., Narmanlioglu H.K., Zia-Ul-Haq M. (2014). Physicochemical and antioxidant characteristics of fruits of cape gooseberry (*Physalis peruviana* L.) from Turkey. Oxid. Commun..

[4] Engin S.P., Mert C. (2020). The effects of harvesting time on the physicochemical components of aronia berry. Turk. J. Agric. For..

[5] Gecer M.K., Kan T., Gundogdu M., Ercisli S., Ilhan G., Sagbas H.I. (2020). Physicochemical characteristics of wild and cultivated apricots (*Prunus armeniaca* L.) from Aras valley in Turkey. Genet. Resour. Crop Evol..

[6] Khan N., Fatima F., Haider M.S., Shazadee H., Liu Z., Zheng T., Fang J. (2019). Genome-Wide Identification and expression profiling of the polygalacturonase (PG) and pectin methylesterase (PME) genes in grapevine (*Vitis vinifera* L.). Int. J. Mol. Sci..

[7] Sayinci B., Ercisli S., Ozturk I., Eryilmaz Z., Demir B. (2012). Determination of size and shape in the ‘Moro’ blood orange and ‘Valencia’ sweet orange cultivar and its mutants using image processing. Not. Bot. Horti Agrobot. Cluj-Napoca.

[8] Zhang S., Hu J., Zhang C.F., Guan Y.J., Zhang Y. (2007). Genetic analysis of fruit shape traits at different maturation stages in sponge gourd. J. Zhejiang Univ. Sci. B.

[9] Goddard M.E., Hayes B.J. (2007). Genomic selection. J. Anim. Breed. Genet..

[10] Sayıncı B., Kara M., Ercişli S., Duyar Ö., Ertürk Y. (2015). Elliptic Fourier analysis for shape distinction of Turkish hazelnut cultivars. Erwerbs-Obstbau.

[11] Feldmann M.J., Hardigan M.A., Famula R.A., López C.M., Tabb A., Cole G.S., Knapp S.J. (2020). Multi-dimensional machine learning approaches for fruit shape phenotyping in strawberry. GigaScience.

[12] Zhang C., Fan X., Liu C., Fang J. (2021). Anatomical berry characteristics during the development of grape berries with different shapes. Hortic. Plant J..

[13] Ercisli S., Sayinci B., Kara M., Yildiz C., Ozturk I. (2012). Determination of size and shape features of walnut (*Juglans regia* L.) cultivars using image processing. Sci. Hortic..

[14] He J.Q., Harrison R.J., Li B. (2017). A novel 3D imaging system for strawberry phenotyping. Plant Methods.

[15] Demir B., Sayinci B., Sümbül A., Yaman M., Yildiz E., Çetin N., Karakaya O., Ercişli S. (2020). Bioactive compounds and physical attributes of *Cornus mas* genotypes through multivariate approaches. Folia Hortic..

[16] Sayıncı B., Ercişli S., Akbulut M., Şavşatlı Y., Baykal H. (2015). Determination of shape in fruits of cherry laurel (*Prunus laurocerasus*) accessions by using Elliptic Fourier analysis. Acta Sci. Pol. Hortoru..

[17] Hayashi A., Tanabata T., Wada T. (2017). A proposal of image analysis system for measuring strawberries. Hort. J..

[18] Osako Y., Yamane H., Lin S.Y., Chen P.A., Tao R. (2020). Cultivar discrimination of litchi fruit images using deep learning. Sci. Hortic..

[19] Maeda H., Akagi T., Tao R. (2018). Quantitative characterization of fruit shape and its differentiation pattern in diverse persimmon (*Diospyros kaki*) cultivars. Sci. Hortic..

[20] Ates F., Coban H., Kara Z., Sabir A. (2011). Ampelographic characterization of some grape cultivars (*Vitis vinifera* L.) grown in South-western region of Turkey. Bulg. J. Agric. Sci..

[21] Bodor P., Baranyai L., Ladányi M., Bálo B., Strever A.E., Isztray G.Y.D., Hunter J.J. (2013). Stability of ampelometric characteristics of *Vitis vinifera* L. cv. ‘Syrah’ and ‘Sauvignon blanc’ leaves: Impact of within-vineyard variability and pruning method/bud load. S. Afr. J. Enol. Vitic..

[22] Gago P., Santiago J.L., Boso S., Villaverde A.V., Orriols I., Martínez M. (2013). Identity of three grapevine varieties from a rediscovered viticulture region in northwest Spain. J. Int. Sci. Vigne Vin..

[23] Eyduran S.P., Akin M., Ercisli S., Eyduran E., Maghradze D. (2015). Sugars, organic acids, and phenolic compounds of ancient grape cultivars (*Vitis vinifera* L.) from lgdir province of Eastern Turkey. Biol. Res..

[24] Isci B., Altindisli A. (2017). Ampelographıc characterızatıon of Turkish indigenous grape accessions and European cultivars (*Vitis vinifera* L.). Int. J. Agric. Environ. Food Sci..

[25] Khalil S., Tello J., Hamed F., Forneck A. (2017). A multivariate approach for the ampelographic discrimination of grapevine (*Vitis vinifera*) cultivars: Application to local Syrian genetic resources. Genet. Res. Crop Evol..

[26] Biniari K., Stavrakaki M. (2018). Genetic study of native grapevine varieties of northern, western and central Greece with the use of ampelographic and molecular methods. Not. Bot. Horti Agrobo..

[27] Vesna M., Morata A., Loira I. (2019). Ampelographic and genetic characterization of Montenegrin grapevine varieties. Advances in Grape and Wine Biotechnology.

[28] Crupi P., Gasparro M., Caputo A.R. (2021). Classification of wine grape biotypes according to their variety and sanitary condition by fingerprinting untargeted analysis. Nat. Prod. Res..

[29] OIV (2001). Organisation Internationale de la Vigne et du Vin. OIV Descriptor List for Grape Varieties and Vitis Species.

[30] Kok D., Bal E., Celik S. (2013). Influences of various canopy management techniques on wine grape quality of *V. vinifera* L. cv. Kalecik Karası. Bulg. J. Agric. Sci..

[31] Lamine M., Zemni H., Ziadi S., Chabaane A., Melki I., Mejri S., Zoghlami N. (2014). Multivariate analysis and clustering reveal high morphological diversity in Tunisian autochthonous grapes (*Vitis vinifera*): Insights into characterization, conservation and commercialization. J. Int. Sci. Vigne. Vin..

[32] Ashwini S., Hipparagi K., Patil D., Jagadeesh S.L., Suma R., Arun K. (2016). Impact of canopy management on growth and yield of wine grapes under northern dry zone of Karnataka. Bioscan.

[33] Bioversity International (2007). Bioversity International Guidelines for the Development of Crop Descriptor Lists.

[34] Wycislo A.P., Clark J.R., Karcher D.E. (2008). Fruit shape analysis of *Vitis* using digital photography. HortScience.

[35] Bodor P., Somogyi E., Baranyai L., Lazar J., Balo B. (2020). Analysis of the grapevine (*Vitis vinifera* L.) berry shape by using elliptic Fourier descriptors. Prog. Agric. Eng. Sci..

[36] Kara M., Sayıncı B., Elkoca E., Öztürk İ., Özmen T.B. (2013). Seed size and shape analysis of registered common bean (*Phaseolus vulgaris* L.) cultivars in Turkey using digital photography. J. Agric. Sci..

[37] Kara M. (2017). Biyolojik Ürünlerin Fiziksel Özellikleri (Tarımsal Ürün ve Gıdaları İçerir). I.

[38] Mohsenin N.N. (1986). Physical Properties of Plant and Animal Materials.

[39] Iwata H., Ukai Y. (2002). SHAPE: A computer program package for quantitative evaluation of biological shapes based on elliptic Fourier descriptors. J. Hered..

[40] Sayıncı B. (2016). Detection of manufacturing defects on orifice geometry of polyacetal (POM) nozzle discs by using the elliptic fourier descriptors. J. Agric. Fac. Bursa Uludağ Univ..

[41] Neto J.C., Meyer G.E., Jones D.D., Samal A.K. (2006). Plant species identification using Elliptic Fourier leaf shape analysis. Comput. Electron. Agric..

[42] Özkan-Koca A. (2012). Ortadoğu’da yayılış gösteren Apis mellifera L. (Hymenoptera: Apidae) Alttürlerinin Geometrik Morfometri Yöntemiyle Analizi. Ph.D. Thesis.

[43] Abiri K., Rezaei M., Tahanian H., Heidari P., Khadivi A. (2020). Morphological and pomological variability of a grape (*Vitis vinifera* L.) germplasm collection. Sci. Hortic..

[44] Khadivi-Khub A., Salimpour A., Rasouli M. (2014). Analysis of grape germplasm from Iran based on fruit characteristics. Braz. J. Bot..

[45] Vafaee Y., Ghaderi N., Khadivi A. (2017). Morphological variation and marker-fruit trait associations in a collection of grape (*Vitis vinifera* L.). Sci. Hortic..

[46] Ekhvaia J., Akhalkatsi M. (2010). Morphological variation and relationships of Georgian populations of *Vitis vinifera* L. subsp. *Sylvestris* (C.C. Gmel.). Flora.

[47] Leão P.C.S., Cruz C.D., Motoike S.Y. (2011). Genetic diversity of table grape based on morphoagronomic traits. Sci. Agric..

[48] Esgici R., Özdemir G., Pekitkan G., Eliçin K., Öztürk F., Sessiz A. (2017). Engineering properties of the Şire grape (*Vitis vinifera* L. Cv.). Sci. Papers Ser. B Hortic..

[49] Abu-Zahra T. (2010). Berry size of Thompson seedless as influenced by the application of Gibberellic acid and cane girdling. Pak. J. Bot..

[50] Barbagallo M.G., Guidoni S., Hunter J.J. (2011). Berry size and qualitative characteristics of *Vitis vinifera* L. cv. Syrah. S. Afr. J. Enol. Vitic..

[51] Kose B. (2014). Effect of rootstock on grafted grapevine quality. Eur. J. Hortic. Sci..

[52] Bart-Plange A., Dzisi K.A., Ampah J. (2012). Effect of drying on selected physical properties of “Asontem” cowpea variety. Int. Sch. Res. Netw. ISRN Agron..

[53] Gundogdu M., Ozrenk K., Ercisli S., Kan T., Kodad O., Hegedus A. (2014). Organic acids, sugars, vitamin C content and some pomological characteristics of eleven hawthorn species (*Crataegus* spp.) from Turkey. Biol. Res..

[54] Gecer M.K. (2020). Biochemical content in fruits of peach and nectarine cultivars. Turk. J. Agric. For..

[55] Rouphael Y., Colla G. (2005). Growth, yield, fruit quality and nutrient uptake of hydroponically cultivated zucchini squash as affected by irrigation systems and growing seasons. Sci. Hortic..

[56] Zia-Ul-Haq M., Ahmad S., Qayum M., Ercisli S. (2013). Compositional studies and antioxidant potential of *Albizia lebbeck* (L.) Benth. Pods and seeds. Turk. J. Biol..

[57] Bujdosó G., Cseke K. (2021). The Persian (English) walnut (*Juglans regia* L.) assortment of Hungary: Nut characteristics and origin. Sci. Hortic..

[58] Kök D., Bal E., Bahar E. (2017). Physical and biochemical traits of selected grape varieties cultivated in Tekirdağ, Turkey. Int. J. Sustain. Agric. Manag. Inform..

[59] Serce S., Ozgen M., Torun A.A., Ercisli S. (2010). Chemical composition, antioxidant activities and total phenolic content of *Arbutus andrachne* L. (Fam. Ericaceae) (the Greek strawberry tree) fruits from Turkey. J. Food Compos. Anal..

[60] Karatas N., Sengul M. (2020). Some important physicochemical and bioactive characteristics of the main apricot cultivars from Turkey. Turk. J. Agric. For..

[61] Kaskoniene V., Bimbiraite-Surviliene K., Kaskonas P., Tiso N., Cesoniene L., Daubaras R., Maruska A.S. (2020). Changes in the biochemical compounds of *Vaccinium myrtillus*, *Vaccinium vitis-idaea*, and forest litter collected from various forest types. Turk. J. Agric. For..

